# Accelerated Accumulation of γ-Aminobutyric Acid and Modifications on Its Metabolic Pathways in Black Rice Grains by Germination under Cold Stress

**DOI:** 10.3390/foods12061290

**Published:** 2023-03-17

**Authors:** Yingjie Yu, Min Li, Chunxiao Li, Meng Niu, Huilong Dong, Siming Zhao, Caihua Jia, Yan Xu

**Affiliations:** 1 Key Laboratory of Environment Correlative Dietology (Ministry of Education), College of Food Science and Technology, Huazhong Agricultural University, Wuhan 430070, China; 2 Guangxi Yangxiang Co., Ltd., Guigang 537100, China

**Keywords:** γ-aminobutyric acid, cold stress, germination, black rice, metabolic pathway

## Abstract

Germination can increase γ-aminobutyric acid (GABA) accumulation in grains, but the combined effects of germination and other external stress on rice grains have been little studied. In this investigation, enhanced accumulation of GABA and modification of its metabolic pathways in black rice grains were investigated during germination under cold stress. The combination of cold stress and germination resulted in a greater accumulation of GABA than germination alone. The treatment of cold stress at 0 °C for 1 h and germination for 72 h induced a maximum GABA content of 195.64 mg/100 g, 51.54% higher compared to the control, which was superior to any other treatment. We modified the metabolism of the GABA shunt to the orientation of GABA synthesis, in which the activity of glutamic acid decarboxylase and protease were stimulated. The total content of free amino acid indicated an upward trend as germination prolonged. The degradation of polyamines was partly promoted due to elevated diamine oxidase and polyamine oxidase activity, but the activity of amino-aldehyde dehydrogenase for the direct synthesis of GABA in the pathway was suppressed. The result implied that the GABA shunt might play a major role in enhancing GABA accumulation induced by cold stress and germination rather than the polyamines degradation pathway. This investigation provides a practical reference for GABA accumulation by germination under cold stress and a theoretical basis for the possible mechanism underlying the accelerating action.

## 1. Introduction

Rice is one of the most widely eaten grains in the world [1]. Rice grains come in many colors; thereinto, black rice is regarded as an excellent choice for nutritious diets [2]. Black rice possesses plenty of physiologically active substances, such as flavonoids, anthocyanins, amino acids, vitamin E, dietary fiber, and minerals [3]. It also has a higher content of protein than regular refined rice. Black rice has become more and more popular in the community that is trying to lose weight or is fond of fitness.

As one kind of free amino acid, γ-aminobutyric acid (GABA), is considered an inhibitory neurotransmitter in humans, which is critical in relieving some symptoms, such as insomnia and anxiety; it has been also related to many neurological control behaviors, such as motor learning ability [4,5,6,7]. The human body cannot produce enough GABA to meet metabolic demands. In order to maintain body operation, extra GABA needs to be supplemented from diets [6]. Plants, particularly grains that are commonly utilized as staple foods, are ideal sources of GABA supplementation. However, the content of GABA is roughly 0.03–3.35 mg/g in grains, which is quite low for dietary intake. As a result, several studies have been conducted to enhance GABA content for the last few years. The promising techniques included controlled germination, environmental stress, and microbial fermentation.

Controlled germination has attracted a lot of attention in recent years because of its ability to enhance the nutritional values of grains, such as wheat, brown rice, oat, and quinoa. Germination, including preharvest sprouting and controlled germination, is a biological process that occurs when endogenous enzymes are activated [8]. Compared with preharvest sprouting, controlled germination is regarded as a more reliable technique because of the higher replicability and stability. Most studies related to the utilization of germination on grains mainly focus on the changes in enzyme activity and compositional alterations. The activities of α-amylase, protease, phytase, and glutamic acid decarboxylase (GAD) in brown rice could be raised after a 24 h or 72 h germination [9,10,11]. The contents of many physiological active substances, such as GABA, linoleic acid, γ-oryzanol, and vitamin E, were also enhanced [12].

Several investigations have reported that GABA content could be significantly increased during germination [13,14,15,16,17]. As for the underlying mechanism of GABA enhancement induced by germination, grains have extensive defense mechanisms that allow them to respond to external biotic and abiotic stress. These stress reactions begin with signal perception and end with a range of responses [18]. As an essential substance in grains, GABA connects the basic metabolism and signal transductions and plays a critical role in the responses to external stress [19]. Grains synthesize GABA mostly through the GABA shunt, which involves glutamate decarboxylation reactions. It also can be synthesized through the oxidative destruction of polyamines [20]. Meanwhile, the synthesis pathways of GABA are susceptible to external environmental changes. As a result, GABA can be enriched through the modifications of its metabolic pathways by exerting external stress in a regulated manner, such as controlled germination.

Cold stress is one of the environmental changes that usually occur in plants. Recently, some studies have reported that freezing or freeze–thaw treatment could accelerate the synthesis of GABA in grains, mainly by inducing cell membrane tissue rupture and solute aggregation [21,22]. However, the modifications in the metabolic pathways of GABA affected by cold stress are still not clear. What is more, the effects of a combination of various external stresses on GABA enrichment have been little investigated so far. Therefore, this study was expected to interpret the enrichment of GABA in black rice and the modifications on its metabolic pathway induced by germination under cold stress. The investigation provides valuable information for the development of GABA-enrichment techniques and GABA-enriched products.

## 2. Materials and Methods

### 2.1. Materials

Black rice used was a type of japonica glutinous rice with a 170-day growing cycle that was grown in Wuchang City, Heilongjiang Province, China. It was harvested in September 2021 and provided by Beijing Golden Harvest Green Source Agricultural Technology Co. (Beijing, China). All reagents used in this study were analytical grade. The effective chlorine content of sodium hypochlorite solution was greater than 14%.

### 2.2. Controlled Germination

**Figure 1:**
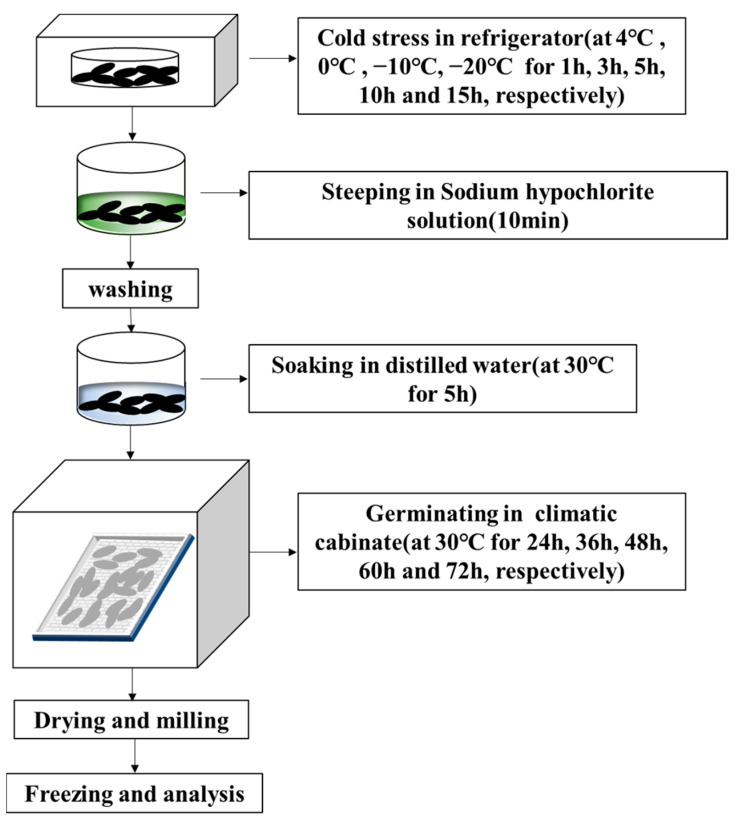
Flow chart of the germination under cold stress.

As seen in Figure 1, controlled germination was carried out by first steeping 50 g of rice grains in 0.1% (*v*/*v*) sodium hypochlorite solution for 10 min and washing 3 times with tap water. Then, rice grains were soaked in 100 mL distilled water at 30 °C for 5 h, spread on a tray with 3 layers of gauze, and incubated for 24–72 h at 30 °C and 90% relative humidity in a climate chamber (HP300GS-LED, Wuhan Ruihua Instrument Equipment Co., Ltd., Wuhan, China). During germination, rice grains were watered daily to keep them moist. The germinated rice grains were dried for 2 h in a blast drier at 40 °C, then milled and finally preserved at 4 °C in a sealed bag.

### 2.3. Cold Stress Treatment and Experimental Design

1. Before germination, black rice grains were placed in a refrigerator at 4, 0, −10, and −20 °C for 1, 3, 5, 10, and 15 h, respectively. The GABA content was measured after 24 h of germination.
2. To investigate the influence of germination time (24, 36, 48, 60, and 72 h) on the content of GABA that had been subjected to cold stress, the stress time with the maximum GABA production at each temperature was chosen.
3. For follow-up investigations, the sample with excellent GABA accumulation after Step 1 or 2 was chosenand germinated black rice grains that had not been subjected to cold stress were utilized as a control.

### 2.4. Determination of GABA Content

To obtain GABA extract, black rice grains were first milled, then 0.5 g of rice flour was accurately weighed and mixed with 20 mL of 60% ethanol solution. The suspension was extracted at 70 °C in a water bath for 2 h and centrifugated at 8000× *g* for 10 min, then 10 mL of the supernatant was aspirated and added with 1 g of activated carbon for 30 min of discoloration. The solution was filtered through a 0.45 μm microfiltration membrane to obtain GABA extract. GABA extract (0.5 mL) was placed in a 10 mL centrifuge tube and added with 0.2 mL of 0.2 mol/L boric acid (pH = 9.0), 1 mL of 6% phenol, 0.4 mL of 10% sodium hypochlorite, and 2 mL of 60% ethanol solution. The mixed solution was set in a boiling water bath for 10 min, then an ice bath for another 20 min. The absorbance value of the solution was measured at 645 nm with an ultraviolet spectrophotometer (UV2600A, Unico, Shanghai, China).

### 2.5. Measurement of Glutamate Decarboxylase (GAD) Activity

GAD was extracted with 50 mmol/L phosphate solution (including 0.2 mmol/L pyridoxal phosphate, 2 mmol/L EDTA, 0.2% (*v*/*v*) β-mercaptoethanol, and 0.15 mol/L NaCl, pH = 5.7). After centrifugation at 10,000× *g* for 15 min, 0.3 mL of supernatant was added to 0.2 mL of 50 mmol/L phosphate buffer (including 0.2 mmol/L pyridoxal phosphate and 100 mmol/L L-glutamic acid, pH = 5.7). The reaction was carried out at 40 °C for 120 min, and distilled water was set as a blank control. The amount of GABA produced was determined using the method described in Section 2.4, and one enzyme activity unit was defined as the amount of enzyme that produces one μmol of GABA per minute [23].

### 2.6. Cytochemical Localization of Ca^2+^ in Germ Cells

A 1 cubic millimeter germ tissue block from the embryo axis was placed into a glutaraldehyde fixative containing 2% potassium pyroantimonate and stored overnight at 4 °C. The sample was washed 3 times with a dipotassium hydrogen phosphate buffer (containing 2% potassium pyroantimonate, pH = 7.6) every 30 min. Then, the sample was transferred into 1% osmic acid (including 2% potassium pyroantimonate pH = 7.6) and stored overnight at 4 °C. After washing twice with 0.1 mol/L phosphate buffer for every 30 min, the sample was dehydrated successively with 50% ethanol and 90% ethanol once, and 100% ethanol and 100% propylene oxide three times. Then, dehydrated sample was embedded in the embedding solution and the embedded material was polymerized in an oven at 37 °C and 45 °C for 12 h, respectively, and finally at 60 °C for 48 h. The material was trimmed and sectioned using an ultrathin sectioning machine (EM UC7, Leica, wetzlar, Germany), and the sections were stained with 3% uranyl acetate–lead citrate dye for 30 min and photographed using a transmission electron microscope (HT7700, Hitachi, Tokyo, Japan). The Ca^2+^ was generated as calcium pyroantimonate in the presence of potassium pyroantimonate, which appeared as black particles in the electron microscopic field.

### 2.7. Measurement of γ-. Aminobutyric Acid Aminotransferase (GABA-T) Activity

Crude GABA-T was extracted with prechilled 0.01 mol/L potassium phosphate buffer (containing 200 mL/L glycerol, 1.30 g/L TritonX-100, 0.10 mmol/L glutathione, 0.10 mol/L pyridoxal phosphate, 1.00 mmol/L Na_2_EDTA, pH = 6.8). GABA-T activity was determined by calculating the production of nicotinamide adenine dinucleotide (NADH, reduced forms). The absorbance value was measured at 340 nm after 15 mL of crude GABA-T was added to 2850 mL of sodium phosphate buffer (containing 1.0 mmol/L NAD^+^, pH = 8.75) and maintained at 30 °C for 30 min. One μmol NADH produced per minute was set as one enzyme activity unit [24].

### 2.8. Measurement of Protease Activity

Crude protease was extracted with a buffer of 0.1 mol/L disodium hydrogen phosphate citrate (pH = 6.0). Protease activity was measured by mixing 1 mL enzyme extract and 1 mL of 2% (*w*/*v*) casein solution, incubating the mixture at 40 °C for 30 min and terminating the reaction (90 °C for 5 min). After cooling, 2 mL of 0.4 mol/L trichloroacetic acid was added to the reaction mixture, and 1 mL of the supernatant was pipetted into a centrifuge tube after centrifuging at 3500× *g* for 10 min. Then, 5 mL of 0.4 mol/L sodium carbonate and 1 mL of 1.0 mol/L Folin reagent were added, and the reaction was carried out at 40 °C for 20 min. Protease activity was determined by calculating the increase in the absorbance at 660 nm. One microgram of tyrosine produced per minute was defined as one enzyme activity unit [25].

### 2.9. Determination of Protein Content and Amino Acids

Protein content in rice grains was determined according to AACC international method 46–12.01. As for amino acid quantifications, 0.50 g of rice flour was mixed with 10 mL of 10% (*v*/*v*) sulfosalicylic acid and shaken for 2 h. After centrifuging at 10,000× *g* for 10 min, 1 mL of supernatant was diluted with a certain ratio and filtered with a 0.45 μm filter membrane. The amino acid content was quantified by an amino acid autoanalyzer (A300, MembraPure Gmbh, Berlin, Germany). The content of proline was measured at 440 nm, while the content of other amino acids was measured at 570 nm.

### 2.10. Measurement of Diamine Oxidase (DAO) and Polyamine Oxidase (PAO) Activity

Crude DAO and PAO were extracted with a buffer containing 70 mmol/L of potassium phosphate and 30% (*v*/*v*) propanetriol (pH = 6.5). A 250 μL of crude enzyme extract was mixed with 50 μL of 200 U/mL horseradish peroxidase and 1 mL of 70 mmol/L potassium phosphate buffer (containing 50 μL of N, N-dimethylaniline and 20 mg/mL 4-amino antipyrine, pH = 6.5). The mixture was kept at 37 °C for 5 min, and 50 μL of 50 mmol/L putrescine (for DAO) or 50 μL of 50 mmol/L spermidine (for PAO) was added. The enzyme activity (one unit) was calculated by a change of 0.001 per minute of the absorbance at 470 nm [26].

### 2.11. Determination of Polyamines Content

First, 500 μL of polyamines standard was mixed with 5 mL of 2 mol/L sodium hydroxide and 300 μL of benzoyl chloride. Then, the mixture was kept at 37 °C for 30 min and 10 mL saturated sodium chloride and 10 mL anhydrous ether were added. After shaking for 120 min and standing for 1 min, the solution was concentrated with high pure nitrogen, and the residue was dissolved in 1.0 mL of methanol and filtered by a 0.45 μm filter membrane. Meanwhile, rice flour was extracted with 5% perchloric acid in an ice bath for 60 min. After centrifuging at 10,000× *g* for 15 min, resulting supernatant was benzoylated by the above methods as used for polyamine standards. The polyamines content was determined by high-performance liquid chromatography (LC-20AT, AB Sciex, Kyoto, Japan) using an external standard method. Chromatographic conditions were mobile phase A (methanol): mobile phase B (water) = 6:4, flow rate: 0.7 mL/min, column: XBridge C18 column (4.6 × 250 mm), column temperature: 37 °C, detection wavelength: 254 nm.

### 2.12. Measurement of Amino-Aldehyde Dehydrogenase (AMADH) Activity

Crude AMADH was extracted with a buffer of 0.1 mol/L potassium phosphate (including 5 mmol/L dithiothreitol, 5 mmol/L ethylenediaminetetraacetic acid, and 10% (*w*/*v*) sucrose, pH = 7.0). A total of 250 μL of crude enzyme extract was mixed with 1 mL of 100 mmol/L Tris-HCl solution (containing 1 mmol/L NAD^+^, 0.2 mmol/L phenazine methosulfate, and 0.8 mmol/L nitrogen blue tetrazolium). After centrifugation, 150 μL of supernatant was mixed with 50 μL of 1 mmol/L 1-Amino-3,3-diethoxypropane, and the enzyme activity (one unit) was calculated by a change of 0.01 per minute of the absorbance at 340 nm [27].

### 2.13. Statistical Analysis

All measurements were performed at least in triplicate. Statistical analyses were performed with SPSS using LSD (least significant differences). Results with *p*-values of less than 0.05 were considered significant by Duncan’s tests.

## 3. Results and Discussion

### 3.1. GABA Content

**Figure 2:**
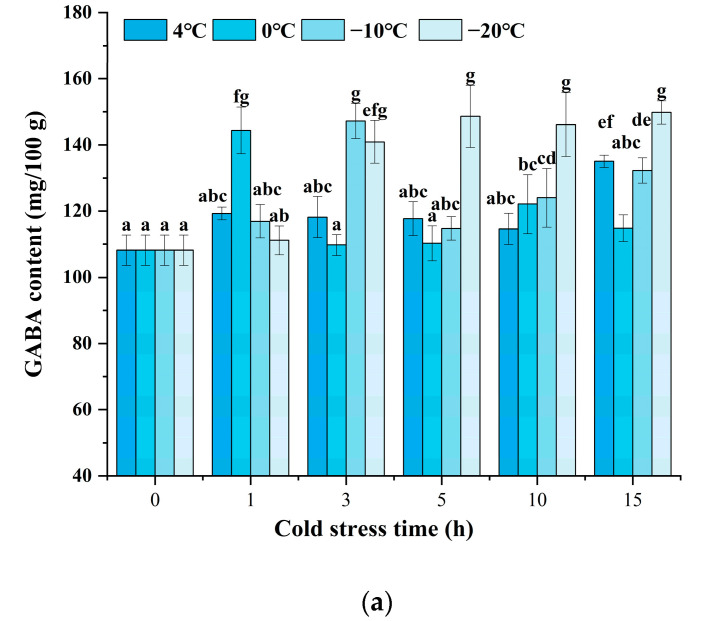

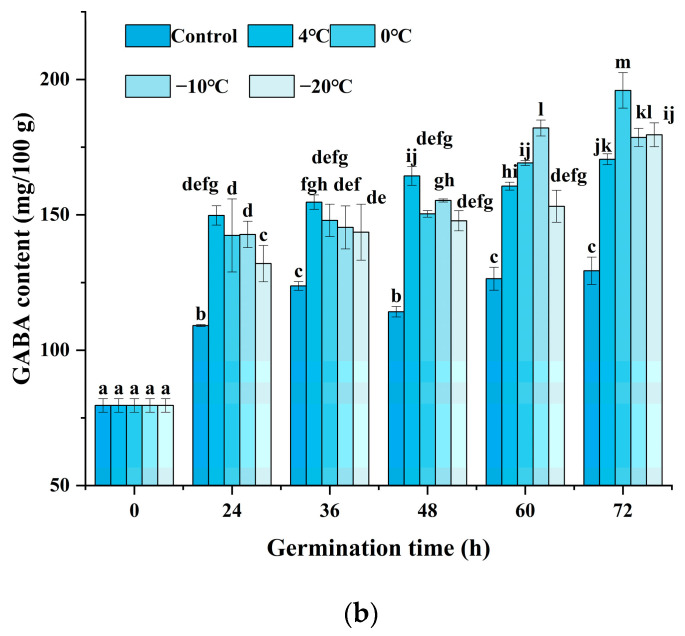
Change in GABA content influenced by cold stress and germination: (**a**) represents the change in GABA content influenced by cold stress temperatures 4 °C, 0 °C, −10 °C, and −20 °C and subjected to cold stress at 4 °C, 0 °C, −10 °C, and −20 °C, respectively, and germinated for 24 h; (**b**) represents the change in GABA content influenced by cold stress temperature and germination time; control, germinated for 0–72 h without cold stress; 4 °C, subjected to cold stress at 4 °C for 15 h and germinated for 0–72 h; 0 °C, subjected to cold stress at 0 °C for 1 h and germinated for 0–72 h; −10 °C, subjected to cold stress at −10 °C for 3 h and germinated for 0–72 h; −20 °C, subjected to cold stress at −20 °C for 5 h and germinated for 0–72 h. Means with different small letter superscripts are significantly different at *p* < 0.05.

As indicated in Figure 2a, the black rice treated with cold stress showed a higher GABA content at a certain stress time after 24 h of germination. Enhanced accumulation of GABA could be significantly observed, and various cold stress temperatures exhibited different patterns. Under cold stress at 4 °C, the GABA content indicated little prominent change until the cold stress time was increased to 15 h. Under cold stress at 0 °C and −10 °C, the GABA content reached a maximum value when the cold stress time was set at 1 h and 3 h, respectively. Moreover, the content of GABA was enhanced since the cold stress time was raised to 3 h when the temperature was at −20 °C. The results indicated that cold stress could accelerate the accumulation of GABA upon germination, and a low temperature below or equal to 0 °C might induce an obvious increase after 1 to 5 h of treatment. Figure 2b shows that the changes in GABA content are affected by germination time and cold stress temperature. The combination of cold stress and germination induced a significant increase in GABA content during the germination time from 24 h to 72 h compared to germination alone (control). As germination time prolonged, the content of GABA showed an obvious increase at each cold stress temperature either from 36 h or from 48 h. The treatment of cold stress at 0 °C for 1 h and germination for 72 h induced the maximum GABA content of 195.64 mg/100 g, 51.54% higher compared to the control, which was superior to any other treatment.

Environmental stress, such as low temperature, normally causes osmotic stress. As a result, plant cell membranes become disordered, and reactive oxygen species (ROS) can be produced in excess followed by photosynthetic suppression and cell structural damage [28]. Meanwhile, plant metabolism can be accordingly modified, including the production of compatible solutes (e.g., GABA, proline, and polyamines). These compounds stabilize proteins and cellular structures and maintain cell turgor through osmotic adjustment, sustain amino acid homeostasis and carbon and nitrogen balance, as well as redox metabolism to remove excess ROS and restore cellular redox balance [29,30,31]. Therefore, a higher GABA content induced by cold stress was one of the responses of black rice grains to external stress. In addition, germination is a biological process that has been widely regarded to have the function of enhancing GABA accumulation [13]. Our results showed that the combination of cold stress and germination induced an increased GABA content compared to germination alone, which probably implied that the metabolic pathway of GABA could be modified to a larger degree when cold stress was included upon germination than germination alone.

### 3.2. GAD Activity and Distribution of Ca^2+^ in Germ Cells

**Figure 3:**
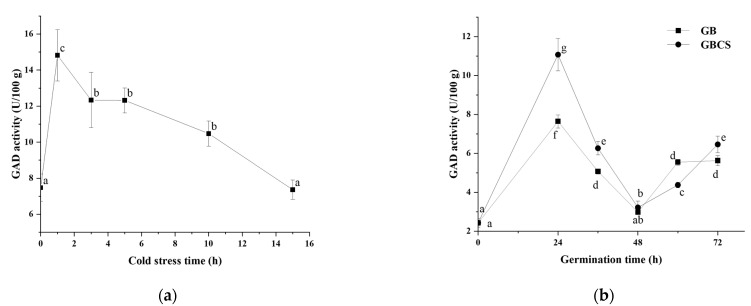
Change in GAD activity influenced by cold stress and germination. GB, germinated black rice grain without cold stress; GBCS, germinated black rice grain under cold stress at 0 °C for 1 h; (**a**) represents the change in GAD activity influenced by cold stress time; (**b**) represents the change in GAD activity influenced by germination time with or without cold stress. Means with different small letter superscripts are significantly different at *p* < 0.05.

Due to the higher efficiency in GABA accumulation, 0 °C of cold stress was chosen to investigate the changes in enzyme activities related to GABA production during germination. GAD is generally regarded as one of the most important enzymes in GABA accumulation; it can decarboxylate glutamate and produce GABA directly [32]. As shown in Figure 3a,b, GAD activity reached the highest value when cold stress was at 1 h and then exhibited a decreasing trend as cold stress time was prolonged. Meanwhile, during germination, GAD activity reached the highest value at 24 h, then declined until 48 h, and finally rebounded at 60 h and 72 h. GBCS showed a higher GAD activity than GB except at the germination time of 48 and 60 h. In order to interpret the relationship between GAD activity and GABA content, correlation analysis was conducted, and the correlation coefficients between the two variables were above 0.9. Our findings indicated that GAD was an important contributor to the enhanced accumulation of GABA by germination under cold stress.

**Figure 4:**
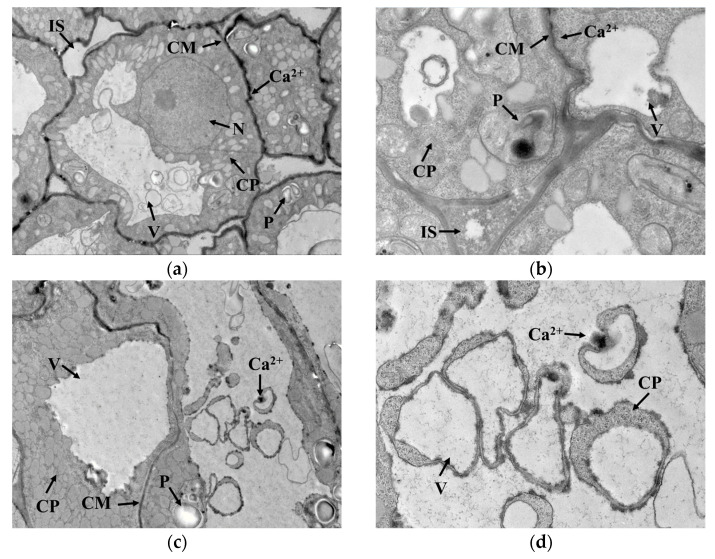

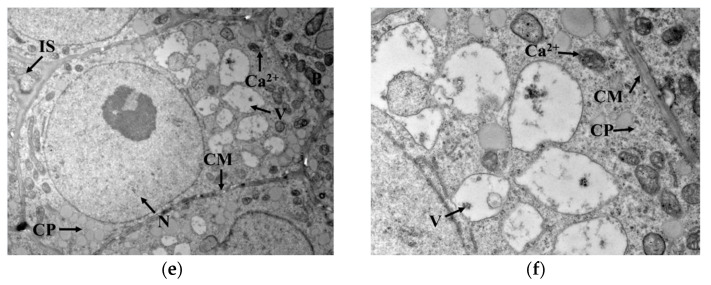
Localization of Ca^2+^ in germ cells: (**a**,**b**) represent the localization of Ca^2+^ in germ cells under no cold stress with magnification at 2000× and 6000×, respectively; (**c**,**d**) represent the localization of Ca^2+^ in germ cells under cold stress at 0 °C for 1 h with magnification at 2000× and 6000×, respectively; (**e**,**f**) represent the localization of Ca^2+^ in germ cells under cold stress at 0 °C for 15 h with magnification at 2000× and 6000×, respectively; N: nucleus; V: vacuole; IS: intercellular space; CP: cytoplasm; P: plastid; CM: cytomembrane. The areas indicated by the arrow with “Ca^2+^”are the precipitates formed by calcium pyroantimonate.

GAD is a water-soluble enzyme with a Ca^2+^/calmodulin (CaM) binding domain on the surface, which is susceptible to Ca^2+^ concentration in the cytoplasm. As seen in Figure 4a,b, Ca^2+^ was mainly distributed outside the cell membrane in germ cells when cold stress was not exerted. Under cold stress, the cell membrane permeability changed, and Ca^2+^ gradually migrated from outside the cell membrane into the cytoplasmic matrix, as indicated in Figure 4c–f. This migration promoted Ca^2+^ concentration in the cytoplasm and contributed to the activation of GAD, thus accelerating the synthesis of GABA. Interestingly, GAD activity showed no upward trend with cold stress time, implying that GAD activity might not be only regulated by Ca^2+^ concentration but also influenced by other conditions in the cytoplasmic matrix that interfered with the binding of Ca^2+^ to CaM.

### 3.3. GABA-T Activity

**Figure 5:**
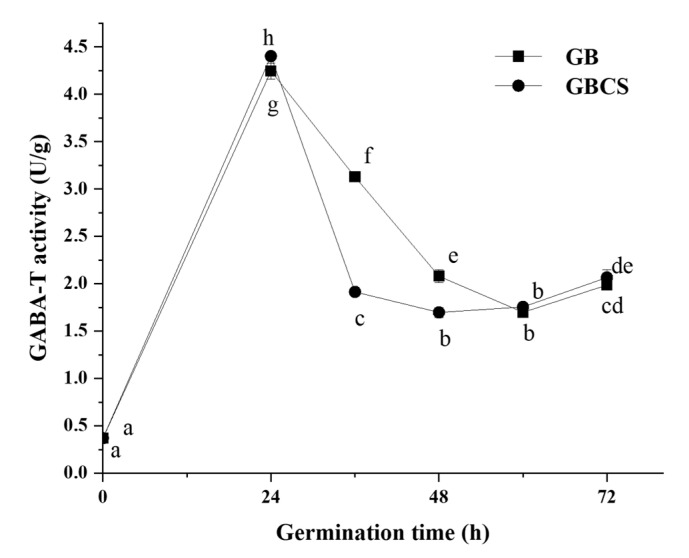
Change in GABA-T activity influenced by cold stress and germination. GB, germinated black rice grain without cold stress; GBCS, germinated black rice grain under cold stress at 0 °C for 1 h. Means with different small letter superscripts are significantly different at *p* < 0.05.

GABA-T is an enzyme that directly degrades GABA in the GABA shunt, and it can be classified into two types in plants: α-ketoglutarate type (GABA-TK) and pyruvate type (GABA-TP). They catalyze the conversion of GABA into succinic semialdehyde by an irreversible transamination process with α-ketoglutaric acid or pyruvate as the receptor; succinic semialdehyde further generates succinic acid, which affects the tricarboxylic acid cycle [33]. GABA-T activity showed little significant change as cold stress time was prolonged from 0 to 72 h. As indicated in Figure 5, GABA-T activity was increased when germination time reached 24 h. GBCS showed a similar pattern with GB except for a slightly smaller activity at the germination time of 36 and 48 h than GB. Theoretically, a reduction in GABA-T activity might enhance the accumulation of GABA since the degradation of GABA was inhibited. The above results indicated that cold stress exerted little change in GABA-T activity. Meanwhile, accelerated accumulation of GABA by germination under cold stress was obviously observed, as shown in Figure 2. Therefore, the results probably implied that cold stress and germination promoted GABA accumulation by activating GAD and accelerating the synthesis of GABA rather than inhibiting GABA degradation. The findings might also indicate that germination under cold stress showed little influence on the entry of succinic acid into the tricarboxylic acid cycle. The modifications in the GABA metabolic pathways by the treatment might not be associated with the tricarboxylic acid cycle.

From the perspective of the GABA shunt pathway, GABA is generally reported as a crucial component in the balance between the carbon and nitrogen pools of cereal cells because it is mainly formed from glutamic acid and can be closely linked to TCAC by the conversion to succinic acid [34]. This pathway begins with the decarboxylation reaction of GAD acting on glutamate in the cytoplasmic matrix to form GABA. The cytosolic GABA is subsequently transported to the mitochondria via a mitochondrial GABA permease known as GABP [35]. In the mitochondria, GABA-T converts GABA to succinic semialdehyde, then succinic semialdehyde is dehydrogenized with a dehydrogenase to produce succinic acid, which enters the TCAC and produces alanine when pyruvate is used as an amino acceptor [36]. GABA is one temporary nitrogen reservoir in the cell. When black rice grains were subjected to cold stress and then germination conditions, the orientation to GABA production in the GABA shunt increased, which was mainly achieved by activated GAD activity. In return, GABA relies on its sensitive control of carbon and nitrogen and acts as a cellular signaling factor to regulate the metabolism and adjust cell behavior to adapt to the environment [37].

### 3.4. Protease Activity, Protein Content, and Free Amino Acids

**Figure 6:**
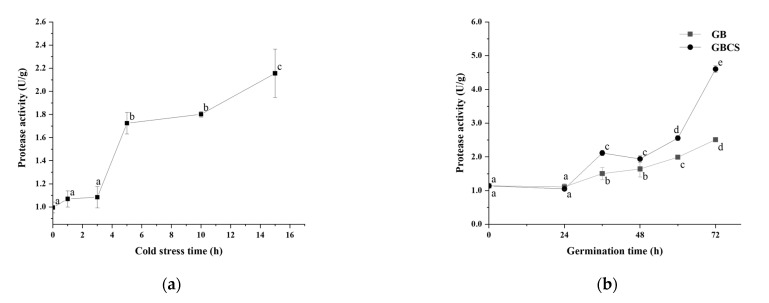
Change in protease activity influenced by cold stress and germination. GB, germinated black rice grain without cold stress; GBCS, germinated black rice grain under cold stress at 0 °C for 1 h; (**a**) represents the change in protease activity influenced by cold stress time; (**b**) represents the change in protease activity influenced by germination time with or without cold stress. Means with different small letter superscripts are significantly different at *p* < 0.05.

**Figure 7:**
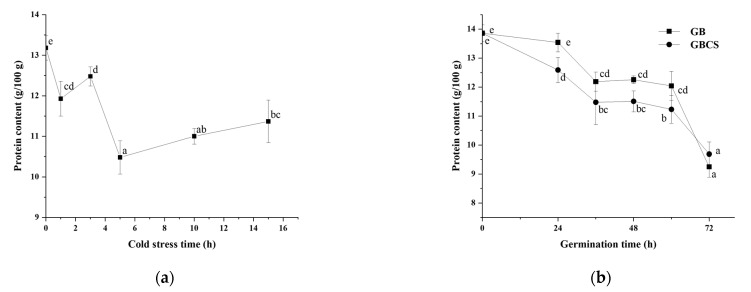
Change in protein content influenced by cold stress and germination. GB, germinated black rice without cold stress; GBCS, germinated black rice under cold stress at 0 °C for 1 h; (**a**) represents the change in protein content influenced by cold stress time; (**b**) represents the change in protein content influenced by germination time with or without cold stress. Means with different small letter superscripts are significantly different at *p* < 0.05.

During germination, activated endogenous proteases break down prolamin and glutelin in the endosperm into peptides and amino acids, which provide a nitrogen source for seed germination. On the other hand, the freshly created amino acids are delivered to the germ and metabolized as nutrients, and the grain cells translated and transcribed them to produce new proteins [25]. As shown in Figure 6a,b, protease activity was activated to a large extent either under cold stress or during germination. Accordingly, the protein content in rice grains declined significantly as the cold stress time prolonged and dropped dramatically during germination from 0 to 72 h, as indicated in Figure 7a,b. The inclusion of cold stress resulted in more reductions in protein content (GBCS vs. GB) during nearly the whole germination period, which was inconsistent with the higher protease activity in GBCS compared to GB, as seen in Figure 6b. The results demonstrated that both cold stress and germination promoted protein degradation; cold stress upon germination resulted in higher protease activity and thus induced more degradation in protein.

As indicated in Table 1, the total content of free amino acids showed an upward trend with increasing germination time either with or without cold stress. Surprisingly, the total amount of free amino acids in GBCS was significantly lower than that in GB when the germination time was 48 and 72 h, which was not consistent with the trend of protein content. The metabolism in grain works as a dynamic and complex system. The phenomenon might be attributed to the accelerated carbon and nitrogen cycle and enhanced metabolic rate of amino acids led by cold stress, and a larger proportion of amino acids were converted to other components, such as organic acid and polyamines. A higher conversion rate thus resulted in a lower total content of amino acids in GBCS [38]. Additionally, glutamate, as a kind of acidic amino acid, is a major substrate for GABA synthesis. Similar to the total content of amino acids, the glutamate content was also increased as germination time prolonged. The lower content in GBCS compared to GB might be due to the greater production of GABA by germination under cold stress.

The content of alanine indicated no obvious change between 24 and 48 h during germination. However, its content was reduced from 114.67 to 99.93 mg/100 g when cold stress was included. In fact, alanine is a byproduct of the GABA shunt that can be catalyzed by GABA-T when pyruvate is employed as the amino acceptor [33]. Thus, the reduction of alanine was probably due to the decrease of GABA-T activity induced by cold stress, as shown in Figure 5. Moreover, proline is regarded as one of the amino acids that are relevant to the plant stress response. It regulates cellular homeostasis, alters mitochondrial activities, influences cell proliferation or death, and stimulates gene expression, all of which are associated with stress recovery [39]. As seen in Table 1, GBCS indicated a higher proline content at respective germination times than GB, which was attributed to the accelerated metabolic pathway of proline led by cold stress to counterbalance the external stress.

### 3.5. DAO and PAO Activity and Polyamines Content

**Figure 8:**
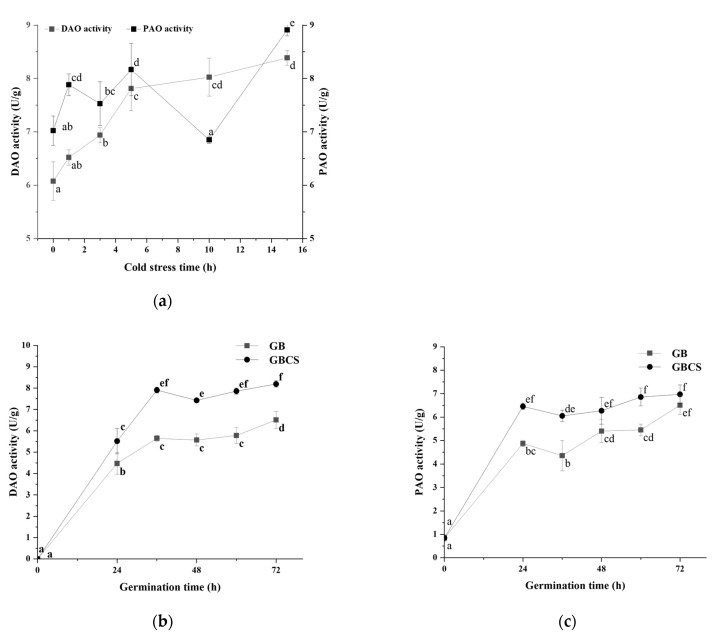
Change in DAO and PAO activity influenced by cold stress and germination. GB, germinated black rice grain without cold stress; GBCS, germinated black rice grain under cold stress at 0 °C for 1 h; (**a**) represents the change in DAO and PAO activity influenced by cold stress time; (**b**) represents the change in DAO activity influenced by germination time with or without cold stress; (**c**) represents the change in PAO activity influenced by germination time with or without cold stress. Means with different small letter superscripts are significantly different at *p* < 0.05.

In the polyamine degradation pathway of GABA, PAO and DAO catalyze the reaction of polyamines to the intermediate product γ-amino butyraldehyde and H_2_O_2_, and γ-amino butyraldehyde is subsequently dehydrogenated with AMADH to produce GABA [40]. As shown in Figure 8a, DAO and PAO activity was stimulated as the stress duration prolonged except for PAO at the stress time of 10 h. Germination also promoted DAO and PAO activity, and longer germination resulted in higher enzymatic activity. Moreover, the combination of cold stress and germination induced a greater increase in enzymatic activity than germination alone, as indicated in Figure 8b,c. These results showed that both cold stress and germination could enhance DAO and PAO activity and thus accelerated the degradation of polyamines. Germination under cold stress resulted in a more elevated degradation of polyamines compared to germination alone.

**Figure 9:**
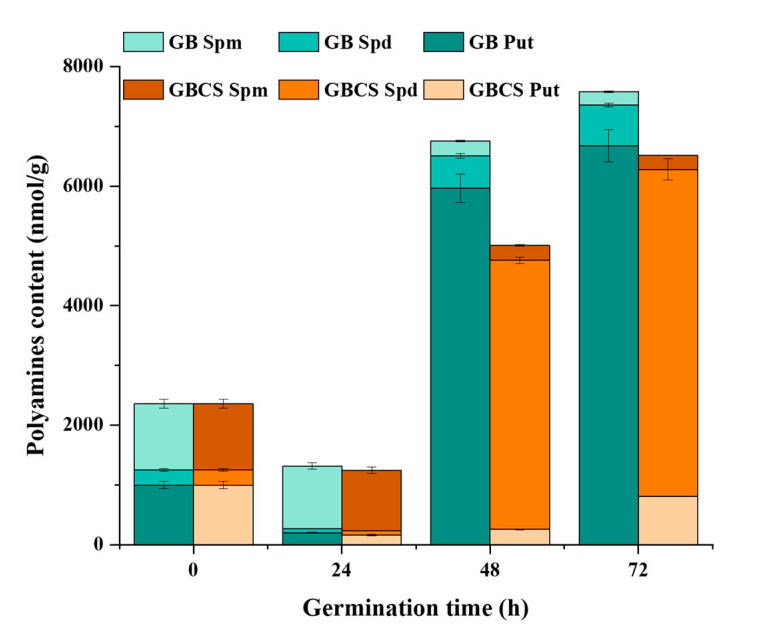
Change in polyamines content influenced by cold stress and germination. GB Spm, Spd, and Put, the content of spermine, spermidine, and putrescine in germinated black rice grain without cold stress, respectively; GBCS Spm, Spd, and Put, the content of spermine, spermidine, and putrescine in germinated black rice grain under cold stress at 0 °C for 1 h, respectively.

Polyamine is a family of biologically active aliphatic nitrogenous bases, such as spermine (Spm), spermidine (Spd), and putrescine (Put) [41]. Polyamines contribute to DNA replication, gene transcription, cell division, and organ development in the plant. They also help maintain ion balance and the stability of antioxidation and the membrane when facing external stress. Figure 9 indicates the changes in Spm, Spd, and Put influenced by cold stress and germination. At 24 h of germination time, a significant decline in polyamines was observed followed by a sharp increase in Put and Spd when germination time reached 48 and 72 h. Thereinto, both Spd and Put content were reduced in GB and GBCS compared to ungerminated grains when germination time was 24 h. However, when germination time reached 48 and 72 h, the content of Put was remarkably increased in GB, while Spd content was significantly enhanced in GBCS. In addition, Spm content was decreased in both GB and GBCS as germination time was prolonged. The sharp increase of Put in GB at 48 and 72 h of germination time might be the response to the germination condition including moisture and temperature, while the notable growth of Spd in GBCS was associated with the response mechanism toward cold stress. According to previous studies, Put could be converted into Spd to resist external stress, which probably explained the increased Spd when cold stress was exerted [41,42,43].

### 3.6. AMADH activity

**Figure 10:**
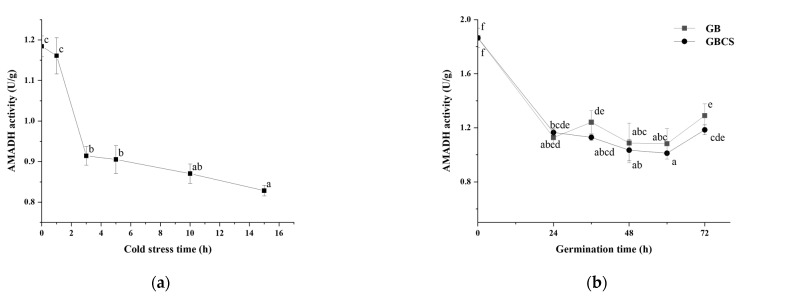
Change in AMADH activity influenced by cold stress and germination. GB, germinated black rice grain without cold stress; GBCS, germinated black rice grain under cold stress at 0 °C for 1 h; (**a**) represents the change in AMADH activity influenced by cold stress time; (**b**) represents the change in AMADH activity influenced by germination time with or without cold stress. Means with different small letter superscripts are significantly different at *p* < 0.05.

In the polyamine degradation pathway, AMADH is the enzyme that catalyzes the synthesis of GABA directly by dehydrogenizing γ-amino butyraldehyde [44]. As shown in Figure 10a, AMADH activity was significantly reduced since the stress time reached 3 h. The result might partly explain the higher GABA content at 1 h of cold stress (0 °C) than at any other stress time. Meanwhile, AMADH activity also decreased since the germination time was 24 h, and GB and GBCS indicated little difference at each germination time, as seen in Figure 10b. From the above data, AMADH activity showed an opposite trend compared to DAO and PAO activity as influenced by cold stress and germination. In light of the polyamine degradation pathway, AMADH is the key enzyme that influences GABA accumulation due to the function of direct synthesis of GABA, although PAO and DAO catalyze the reaction to produce γ-amino butyraldehyde that is the substrate of AMADH to synthesize GABA. The decreased AMADH activity resulting from cold stress and germination probably implied that the polyamine degradation pathway might not contribute to the enhanced accumulation of GABA as much as the GABA shunt pathway, and the GABA shunt might be the pathway that is modified to a larger degree when cold stress and germination were included.

## 4. Conclusions

The combination of cold stress and germination resulted in a greater accumulation of GABA than germination alone. The GABA shunt might play a major role in enhancing GABA accumulation rather than the polyamines degradation pathway as indicated by activated GAD and protease activity, increased free amino acids content, modified Ca^2+^ distribution, and declined AMADH activity. This investigation provides valuable information on the techniques of GABA enrichment and the development of GABA-enriched products.

## Figures and Tables

**Table 1:** Free amino acid contents influenced by cold stress and germination.

Amino Acid (mg/100 g)	NGB	GB-24	GB-48	GB-72	GBCS-24	GBCS-48	GBCS-72
Asp	25.07 ^a^	54.92 ^c^	63.61 ^d^	44.91 ^b^	56.76 ^c^	46.28 ^b^	42.13 ^b^
Thr	4.34 ^a^	32.67 ^c^	36.44 ^c^	43.31 ^d^	27.84 ^b^	25.22 ^b^	25.45 ^b^
Ser	10.62 ^a^	41.82 ^cd^	43.52 ^d^	61.37 ^e^	35.43 ^b^	39.51 ^c^	42.67 ^d^
Glu	30.31 ^a^	71.52 ^c^	102.39 ^e^	112.37 ^f^	61.78 ^b^	87.34 ^d^	96.01 ^e^
Gly	16.29 ^a^	53.55 ^e^	49.90 ^d^	47.69 ^d^	53.55 ^e^	40.73 ^c^	34.73 ^b^
Ala	50.63 ^a^	116.21 ^c^	116.99 ^c^	183.59 ^d^	114.67 ^c^	99.93 ^b^	129.23 ^d^
(Cys)_2_	7.46 ^a^	37.43 ^c^	57.36 ^d^	87.92 ^e^	42.62 ^c^	42.95 ^c^	26.48 ^b^
Val	2.68 ^b^	1.52 ^a^	12.81 ^d^	8.56 ^c^	9.63 ^c^	12.93 ^d^	30.94 ^e^
Met	4.21 ^a^	12.97 ^b^	41.18 ^d^	59.35 ^f^	26.70 ^c^	36.79 ^d^	50.00 ^e^
Ile	3.33 ^a^	15.71 ^b^	30.14 ^d^	51.64 ^f^	22.81 ^c^	29.09 ^d^	36.97 ^e^
Leu	7.34 ^a^	51.24 ^b^	76.05 ^d^	128.82 ^e^	58.89 ^c^	63.94 ^c^	84.73 ^d^
Tyr	3.34 ^a^	25.41 ^b^	37.03 ^c^	61.21 ^d^	23.49 ^b^	27.90 ^b^	36.67 ^c^
Phe	2.47 ^a^	22.10 ^bc^	29.20 ^c^	59.07 ^e^	20.82 ^b^	20.50 ^b^	40.21 ^d^
Lysine-hyd	15.21 ^a^	42.65 ^b^	76.21 ^e^	90.56 ^f^	47.67 ^b^	61.68 ^c^	69.16 ^d^
His	55.51 ^a^	59.55 ^a^	77.58 ^c^	84.75 ^d^	57.91 ^a^	60.31 ^ab^	64.61 ^b^
Arg	18.10 ^a^	68.20 ^c^	81.77 ^d^	100.59 ^e^	64.57 ^bc^	64.74 ^bc^	59.58 ^b^
Pro	7.37 ^a^	25.17 ^b^	27.80 ^b^	46.39 ^d^	29.21 ^bc^	32.99 ^c^	52.80 ^e^
Total	264.29 ^a^	732.66 ^b^	959.96 ^f^	1272.10 ^g^	754.35 ^c^	792.81 ^d^	922.38 ^e^

Note: NGB, ungerminated black rice grain; GB-24, -48, and -72, black rice grain germinated for 24, 48, and 72 h, respectively, without cold stress; GBCS-24, -48, and -72, black rice grain germinated for 24, 48, and 72 h, respectively, under cold stress at 0 °C for 1 h; Asp, asparagine; Thr, threonine; Ser, serine; Glu, glutamine; Gly, glycine; Ala, alanine; (Cys)_2_, cystine; Val, valine; Met, methionine; Ile, isoleucine; Leu, leucine; Tyr, tyrosine; Phe, phenylalanine; Lysine-hyd, lysine hydrochloride; His, histidine; Arg, arginine; Pro, proline. Results are presented as means (n = 3). Means with different small letter superscripts in the same row are significantly different at *p* < 0.05.

## Data Availability

The data presented in this study are available on request from the corresponding author.
